# Syntax-based transfer learning for the task of biomedical relation extraction

**DOI:** 10.1186/s13326-021-00248-y

**Published:** 2021-08-18

**Authors:** Joël Legrand, Yannick Toussaint, Chedy Raïssi, Adrien Coulet

**Affiliations:** 1 Université de Lorraine, CNRS, Inria, LORIA, Nancy, 54000 France; 2 Stanford University, Stanford Center for Biomedical Informatics Research, Stanford, CA USA

**Keywords:** Transfer learning, Deep learning, Biomedical relation extraction

## Abstract

**Background:**

Transfer learning aims at enhancing machine learning performance on a problem by reusing labeled data originally designed for a related, but distinct problem. In particular, domain adaptation consists for a specific task, in reusing training data developedfor the same task but a distinct domain. This is particularly relevant to the applications of deep learning in Natural Language Processing, because they usually require large annotated corpora that may not exist for the targeted domain, but exist for side domains.

**Results:**

In this paper, we experiment with transfer learning for the task of relation extraction from biomedical texts, using the TreeLSTM model. We empirically show the impact of TreeLSTM alone and with domain adaptation by obtaining better performances than the state of the art on two biomedical relation extraction tasks and equal performances for two others, for which little annotated data are available. Furthermore, we propose an analysis of the role that syntactic features may play in transfer learning for relation extraction.

**Conclusion:**

Given the difficulty to manually annotate corpora in the biomedical domain, the proposed transfer learning method offers a promising alternative to achieve good relation extraction performances for domains associated with scarce resources. Also, our analysis illustrates the importance that syntax plays in transfer learning, underlying the importance in this domain to privilege approaches that embed syntactic features.

## Introduction

A bottleneck for training deep learning-based architectures on text is the availability of large enough annotated training corpora. This is especially an issue in highly specialized domains such as those of biomedicine. Transfer Learning (TL) approaches address this problem by leveraging existing labeled data originally designed for related tasks or domains [1]. However, adaptation between dissimilar domains may lead to negative transfer, i.e. transfer that decreases the performance for the target domain. In this article, we apply a TL strategy using the TreeLSTM model for the task of biomedical Relation Extraction (RE). We propose an analysis of the syntactic features of source and target domain corpora to provide elements of interpretation for the improvements we obtained.

**Fig. 1:**
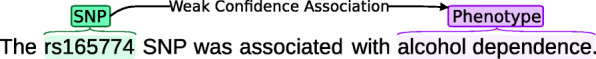
Example of relationship typed as *Weak Confidence Association* between two named entities: a *SNP (single nucleotide polymorphism)* and a *Phenotype*, from the SNPPhenA corpus

Relation Extraction (RE) aims at identifying in raw and unstructured text all the instances of a predefined set of relations between identified entities. A relationship takes the form of an edge between two or more named entities as illustrated in Fig. 1. We are considering here the extraction of typed and binary relationships that consists in, given a set of identified entities, predicting whether there is a relation between pairs of entities, and if so, its type. RE can be seen as a classification task by computing a score for each possible relation type, given a sentence and two identified entities.

Deep learning methods have demonstrated good ability for RE [2], but one of their drawbacks is that, in order to obtain reasonable performances, they generally require a large amount of training data, i.e., text corpora where entities and relationships between them are annotated. The assembly of this kind of domain- and task-specific corpora, such as those of interest in biomedicine, is time consuming and expensive because it involves complex entities (e.g., genomic variations, complex phenotypes), complex relationships (which may be hypothetical, contextualized, negated, *n*-ary) and requires trained annotators. This explains why only few and relatively small (i.e., few hundreds of sentences) corpora are available for some biomedical RE tasks, making these resources particularly valuable. Distinct approaches, such as TL or *distant supervision* [3] have been particularly explored to overcome this limit. With the latter approach, existing relationships available in knowledge- or data-bases are used to enrich the training set, without considering more labeled corpora.

Domain adaptation is a type of TL that allows taking advantage of data annotated for a *source* domain to improve the performances in a related *target* domain [1]. However, even if the source and target domain share the same language (i.e., English), thus a common syntax, TL between domains may lead to negative transfer since specific source domains may use specific vocabularies as well as specific formulations that are inadequate to the target domain. Hence, we need to better understand and characterize what makes a source corpus potentially helpful, or harmful, with regard to a RE task.

The contribution of this paper is twofold. First, we show that, compared to a baseline Convolutional Neural Network (CNN)-based model, a syntax-based model (i.e., the TreeLSTM model) can better benefit from a TL strategy, even with very dissimilar additional source data. We conduct our experiments with two biomedical RE tasks and relatively small associated corpora, SNPPhenA [4] and EU-ADR [5] as target corpora and three larger RE corpora, Semeval 2013 DDI [6], ADE-EXT [7], reACE [8] as source corpora. Second, we propose a syntax-based analysis, using both quantitative criteria and qualitative observations, to better understand the role of syntactic features in the TL behavior.

## Related work

### Deep learning models for relation extraction

Deep learning models, based on continuous word representations have been proposed to overcome the problem of sparsity inherent to NLP [9]. In [10], the authors proposed a unified CNN architecture to tackle various NLP problems traditionally handled with statistical approaches. They obtained state-of-the-art performances for several tasks, while avoiding the hand design of task specific features.

Zeng et al. [2] showed that CNN models can also be applied to RE. In this study, they learn a vectorial sentence representation, by applying a CNN model over word and word position embeddings, which is used to feed a softmax classifier [11]. To improve the performance of RE, authors, such as [12] and [13], consider elements of syntax within the embedding provided to the model.

Beside CNN models that incorporate syntactic knowledge in their embeddings, other approaches proposed neural networks (NN) in which the topology is adapted to the syntactic structure of the sentence. In particular, Recursive Neural Network (RNN) have been proposed to adapt to tree structures resulting from constituency parsing [14, 15]. In this vein, [16] introduced a TreeLSTM, a generalization of LSTM (Long Short-term Memory) network for tree-structured network topologies, which allows processing trees with arbitrary branching factors.

The first model to use RNN for RE was proposed by [17]. The authors introduced a CNN-based model applied on the shortest dependency path between two entities, augmented with a RNN-based feature designed to model subtrees attached to the shortest path. Miwa and Bansal [18] introduced a variant of the TreeLSTM that allows, like the model used in this paper, to take the whole dependency tree into account and not only the shortest path between two entities.

In this paper, we compare two deep learning strategies for RE: (1) the MultiChannel CNN (MCCNN) model [19], which has been successfully applied to the task of protein-protein interaction extraction without using any syntactic feature as input and (2) the TreeLSTM model [16], which is designed for considering dependency trees.

### Transfer learning

TL allows to overcome the lack of training data for a given *target* task by transferring knowledge from *source* data not originally designed for that purpose [1]. One can distinguish *multitask learning* in which performances on a given task are improved using information contained in the training signals of auxiliary related tasks [20] from *domain adaptation* in which only one task is considered but its application domains differ [21]. While the former is a form of inductive transfer in which the auxiliary task introduces an inductive bias during training, the latter is a form of transductive transfer.

Domain adaptation approaches have been proposed for RE, including kernel based methods such as [22] focusing on unsupervised domain adaptation (i.e., without any labeled target data) and deep learning based ones such as [23, 24] focusing on domain adversarial learning (an approach which ensures that the feature distributions over the source and target domains are made similar using an extra domain classifier at train time). Differently, our approach is a case of multi-source domain adaptation (i.e., implying that we have labeled data, both in target and source corpora) and does not involve adversarial training.

Negative transfer occurs when the information learned from a source domain and task has a negative impact on the performances of the target task. Despite the fact that negative transfer is a major issue in TL, to our knowledge only few works have been conducted to overcome this problem [1]. Most of them use a relatedness metrics to select the elements of the source that are the most related to the target. For instance, [25] defined a positive transferability measure that allows removing irrelevant source data. Ge et al. [26] also focused on domain adaptation from multiple sources. They proposed a method to avoid negative learning caused by unrelated or irrelevant source domains, using a weighting mechanism based on a relatedness metrics between the source and target data.

In this work, we experiment with a domain adaptation method on the RE task using the TreeLSTM model, with relatively small biomedical corpora as target corpora and, larger biomedical or general domain corpora as source corpora. We also provide elements of interpretation of the impact of syntactic dependency structures on TL. In this matter, and unlike [25] or [26], the relatedness measures used in this work emphasizes the key role of syntax in TL with TreeLSTM.

## Methods

In this section, we begin with introducing the two compared models, then we present data, i.e. *source* and *target* corpora, and finally, we present the transfer learning strategy and the experimental setting.

### Models

We compare in this article the performances of the MCCNN and TreeLSTM models. Both models compute a fixed-size vector representation for a whole sentence by composing input embeddings. A score is computed for each possible type of relationship (e.g., negative, positive or speculative) between two identified entities. In this subsection, we first introduce the embedding input layer, which is common to both approaches (i.e., MCCNN and TreeLSTM); Then, we detail how each approach composes sequences of embedding in order to compute a unique vectorial sentence representation; Finally, we present the scoring layer, which is common to both approaches.

#### Input layer

Both models are fed with *word embeddings* (i.e., continuous vectors) of dimension *d*_*w*_, along with extra *entity embeddings* of size *d*_*e*_. These embeddings are concatenated to form the input of the model. Formally, given a sentence of *N* words, $w_{1}, w_{2}, \dots, w_{N}$, each word $w_{i} \in \mathcal {W}$ is first embedded in a *d*_*w*_-dimensional vector space by applying a lookup-table operation: $\phantom {\dot {i}\!}LT_{W}(w_{i}) = W_{w_{i}} \ ,$ where the matrix $W \in R^{d_{w} \times |\mathcal {W}|}$ represents the parameters to be trained in this lookup-table layer. The dictionary $\mathcal {W}$ is composed of all the words of the given corpus. Each column $W_{w_{i}} \in R^{d_{w}}$ corresponds to the vector embedding of the *w*_*i*_^*th*^ word in our dictionary $\mathcal {W}$.

Besides, entity embeddings (coming from a simple 3-elements dictionary) enable to distinguish between words which compose either the first entity, the second entity or are not part of any entity. They are respectively called *first entity*, *second entity* and *other* embeddings. Finally, word and entity embeddings are concatenated to form the input corresponding to a given word. Let’s denote *x*_*i*_ the concatenated input corresponding to the *i*^*th*^ word.

#### Composition layers

Both models take the embeddings as input and output a fixed-size representation *r*_*s*_ of size *d*_*s*_, which corresponds to the whole sentence with two identified entities. Accordingly, one sentence with more than two entities will lead to one embedding for each pair of entities. This section details the two models used in this study.

**MCCNN** The MCCNN models applies a variable kernel size CNN to multiple input channels of word embeddings. Inspired by the three-channel RGB image processing models, it considers different embedding channels (i.e., different word embeddings versions for each word) allowing to capture different aspects of input words.

More formally, given an input sequence $x_{1}, \dots, x_{N}$, applying a kernel to the *i*^*th*^ window of size *k* is done using the following formula:

$$C = h\left(\sum_{j=1}^{N-k+1} W \left[x_{i}, \dots, x_{i+k-1}\right]^{j} + b\right) $$

where [ ]^*j*^ denotes the concatenation of inputs from channel *j*, $W \in \mathcal {R}^{(d_{w}+d_{e}) \times d_{h}}$ and $b \in \mathcal {R}^{d_{h}}$ are the parameters, *d*_*h*_ is the size of the hidden layer, *h* is a pointwise non-linear function such as the hyperbolic tangent and *c* is the number of input channels. For each kernel, a fixed size representation $r_{h} \in \mathcal {R}^{d_{h}} $ is then obtained by applying a max-pooling over time (here, the “time” means the position in the sentence).:

$$r_{h} = \max{C} $$

**Fig. 2:**
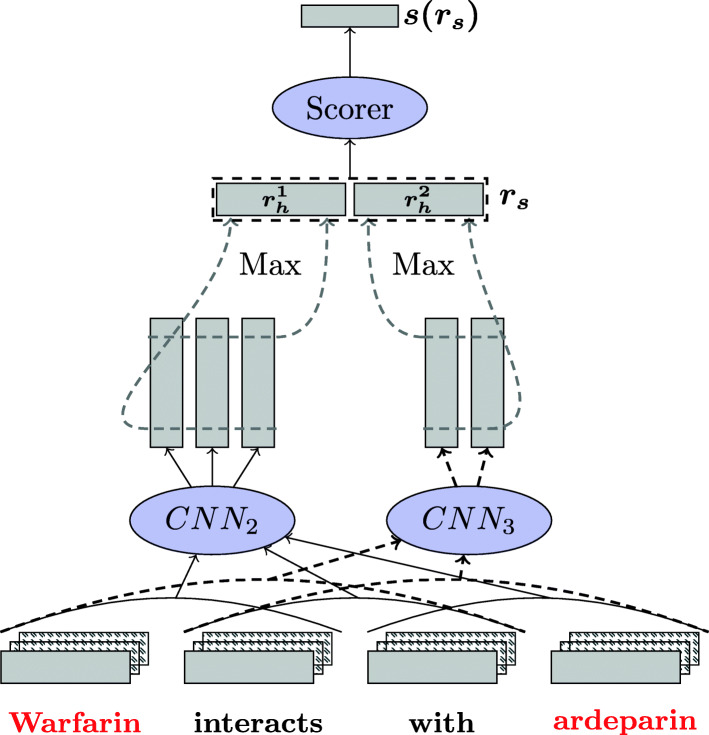
The MCCNN model with three channels, two CNN kernels of size 2 (*CNN*_2_) and 3 (*CNN*_3_). Red words correspond to the entities

We denote *K* the number of kernels with different sizes. A sentence representation $r_{s} \in \mathcal {R}^{d_{s}}$ (with *d*_*s*_=*K*∗*d*_*h*_) is finally obtained by concatenating the output corresponding to the *K* kernels

$$r_{s} = \left[r_{h}^{1}, \dots, r_{h}^{k}\right] \ , $$

where $r_{h}^{k}$ corresponds to the output of the *k*^*th*^ kernel. Figure 2 illustrates the structure of a two-channel CNN, with two kernels of size 2 and 3, on a four-words sentence.

**TreeLSTM** The TreeLSTM model, and more specifically its *Child-Sum* version, [16] processes the dependency tree associated with an input sentence in a bottom-up manner. This model is suitable for processing dependency trees since it handles trees with arbitrary branching factors and no order between children of a node. This is done by recursively processing the nodes of the tree, using at each iteration, the representations of the children of the current node as input. The transition function for a node *j* and a set of children *C*(*j*) can be found in the original paper [16] using $x_{j} \in \mathcal {R}^{d_{w} + d_{e}}$ as input for node *j*.

The transition function for a node *j* and a set of children *C*(*j*) is given by the following set of equations:

$$\begin{array}{*{20}l} \tilde{h}_{t} &= \sum_{k\in C(j)}{ h_{k} }\\ i_{j} &= \sigma \left(W^{(i)} x_{j} + U^{(i)} \tilde{h}_{j} + b^{(i)}\right)\\ f_{jk} &= \sigma \left(W^{(f)} x_{j} + U^{(f)} h_{k} + b^{(f)}\right)\\ o_{j} &= \sigma \left(W^{(o)} x_{j} + U^{(o)} \tilde{h}_{j} + b^{(o)}\right)\\ u_{j} &= \tanh \left(W^{(u)} x_{j} + U^{(u)} \tilde{h}_{j} + b^{(u)}\right)\\ c_{j} &= i_{j} \odot u_{j} + \sum_{k \in C(j)}{f_{jk} \odot c_{k}}\\ h_{j} &= o_{j} \odot \tanh (c_{j}), \end{array} $$

where *σ* denotes the logistic function, ⊙ the element-wise multiplication, $x_{j} \in \mathcal {R}^{d_{w} + d_{e}}$ is the input for node *j*, $h_{k} \in \mathcal {R}^{d_{h}}$ is the hidden state of the *k*^*th*^ child. Each TreeLSTM unit is a collection of vectors: an input gate *i*_*j*_, a forget gate *f*_*jk*_, an output gate *o*_*j*_, a memory cell *c*_*j*_ and hidden state *h*_*j*_. The matrices *W* and *U* and the vectors *b* are the weight and bias parameters to train.

**Fig. 3:**
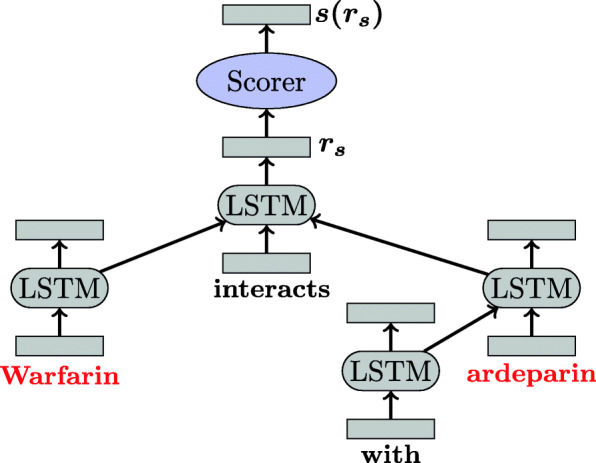
The TreeLSTM model. Each node takes as input the representation of its children. Red words correspond to the entities

The TreeLSTM outputs a sentence representation $r_{s} \in \mathcal {R}^{d_{s}}$ corresponding to the output state *o*_*j*_ of the top tree node (i.e., the *root* node of the dependency tree that spans all the others). Figure 3 illustrates the structure of the TreeLSTM computed for a four-words sentence.

#### Scoring layer

Both the MCCNN and TreeLSTM models output a unique vector representation $r_{s} \in \mathcal {R}^{d_{s}}$ that takes the entire sentence into account, as well as two identified entities. This representation is used to feed a single layer NN classifier, which outputs a score vector with one score for each possible type of relationship. This vector is obtained using the formula:

$$s(r_{s}) = W^{(s)} r_{s} + b^{(s)} $$,

where $W^{(s)} \in \mathcal {R}^{d_{s} \times |S|}$ and $b^{(s)} \in \mathcal {R}^{|S|}$ are the trained parameters of the scorer, |*S*| is the number of possible relation types. The scores are interpreted as probabilities using a softmax layer [11].

### Datasets

We explore how RE tasks that focus on a type of relationship associated with scarce resources may take advantage from larger corpora developed for distinct domains. To this purpose, we selected *(i)* two small *target* biomedical corpora and *(ii)* three larger *source* corpora. Small corpora are SNPPhenA and the EU-ADR corpus composed of annotations of SNP–phenotype relationships for the first and of three different types of relationships related to drug adverse reactions for the second. Large corpora are the SemEval 2013 DDI corpus, the ADE corpus and the reACE corpus. All are publicly available and focus on biomedical relationships, except for the reACE corpus, which is of general domain. Table 1 summarizes the main characteristics of these five corpora and the following subsection details them.

**Table 1:** Main characteristics of our target and source corpora

	Corpus name	Subcorpus	Train Size		Test Size		#Entity Types	#Relation Types
			sent.	rel.	sent.	rel.		
Target	SNPPhenA	–	362	935	121	365	2	3
	EU-ADR	drug-disease	244	176			4	3
		drug-target	247	310	–	–	4	3
		target-disease	355	262			4	3
Source	SemEval	DrugBank	5,675	3,805	973	889	4	4
	2013 DDI	MEDLINE	1,301	232	326	95	4	4
	ADE-EXT	–	5,939	6,701	–	–	2	1
	reACE	–	5,984	2,486	–	–	4	5

Two corpora are divided into subcorpora. The sizes of the training and test corpora are reported in term of number of sentences (sent.) and annotated relationships (rel.). EU-ADR, ADR-EXT and reACE have no proper test corpus

#### Target corpora

**SNPPhenA** [4] is a corpus of abstracts of biomedical publications, obtained from PubMed [27], annotated with two types of entities: *single nucleotide polymorphisms* (SNPs) and *phenotypes*. Relationships between them are annotated and classified in 3 types: *positive*, *negative* and *neutral*. The *neutral* type is used when no relationship is mentioned between two entities, whereas the *negative* is used when a negated relationship is mentioned.

**EU-ADR** [5] is a corpus of PubMed abstracts annotated with *drugs*, *diseases* and drug targets (*proteins/genes* or *gene variants*) entities. It is composed of 3 subcorpora of 100 abstracts each, encompassing annotations of either target-disease, target-drug or drug-disease relationships. Annotated relationships are classified in 3 types: *positive*, *speculative* and *negative associations* (PA, SA and NA respectively). In [28], performances are assessed over the TRUE class, which is composed of the PA, SA and NA types, in contrast with the FALSE class, composed of sentences where two entities co-occur, with no relationship annotated between them.

#### Source corpora

**SemEval 2013 DDI** (Drug-Drug Interaction) [6] consists of texts from DrugBank and MEDLINE annotated with drugs. Drug are categorized in 4 categories: *drug*, *brand*, *group* and *drug_n* (i.e., active substances not approved for human use). Relationships between two drugs are annotated and classified in 4 types: *mechanism*, *effect*, *advice* and *int* (default category, when no detail is provided).

**ADE-EXT** (Adverse Drug Effect corpus, extended) [7] consists of MEDLINE case reports, annotated with *drugs* and *conditions* (e.g., diseases, signs and symptoms), along with untyped relationships between them, when one is mentioned.

**reACE** (Edinburgh Regularized Automatic Content Extraction) [8] consists of English broadcast news and newswire annotated with *organization*, *person*, *fvw* (facility, vehicle or weapon) and *gpl* (geographical, political or location) entities along with relationships between them. Relationships are classified in five types: *general-affiliation*, *organisation-affiliation*, *part-whole*, *personal-social* and *agent-artifact*.

### Training strategy and experimental settings

Our models were trained by minimizing the log-likelihood over the training data. All parameters (weights, biases and embeddings) were iteratively updated via backpropagation for the MCCNN and backpropagation Through Structure [29] for the TreeLSTM. Following a standard practice in deep learning, the transfer learning is done by training models in parallel while using shared representations, as illustrated by [10]. In other terms, for each experiment, the same network, initialized with random weights, is used for each corpus (i.e., same embedding layer and TreeLSTM weights), except for the scorer, which is adapted to each corpus as the number and types of relationships may change. During the training phase, using a standard stochastic gradient descent procedure [30], we randomly pick training sentences from the mixed corpus (i.e., target + one source training corpora). This training procedure is done, starting from different random initialization for each fold of our cross-validation.

Hyper-parameters were tuned using a 10-fold cross-validation by selecting the values leading to the best averaged performance, and fixed for the remaining experiments. Word embeddings were pre-trained on 3.4 million PubMed abstracts (corresponding to all those published between Jan. 1, 2014 and Dec. 31, 2016) using the method described in [31].

**MCCNN model** Following [32] both channels were initialized with pre-trained word embeddings, but gradients were backpropagated only through one of the channels. Hyper-parameters were fixed to *d*_*w*_=100,*d*_*e*_=10,*d*_*h*_=100 for each of the 2 channels, *d*_*s*_=2×*d*_*h*_=200. We used two kernels of size 3 and 5 respectively. We applied a dropout regularization after the embedding layers [33] with a dropout probability fixed to 0.25.

**TreeLSTM model** Dependency trees were derived from parsing trees obtained using the Charniak-Johnson parser trained on GENIA and PubMed data [34]. Hyper-parameters were fixed to *d*_*w*_=100,*d*_*e*_=10,*d*_*h*_=200 and *d*_*s*_=200. We applied a dropout regularization after every TreeLSTM unit and after the embedding layers. The dropout probability was fixed to 0.25. All the parameters are initialized randomly except the word embeddings.

We evaluated performances in terms of precision (P), recall (R) and f-measure (F). For multi-label classifications, we report the macro-average performance^1^. For SNPPhenA, we performed a cross-validation using 10% of the corpus for the validation and the provided test corpus for testing (which is about 30% the size of the training corpus). Because no test corpus is provided with EU-ADR, we performed a 10-fold cross-validation using 10% of the corpus for the validation and 10% for the test of our models.

## Results

This section presents first the results of our transfer learning strategy, and then its comparison with state-of-the-art systems. Finally, we present an analysis of the role of syntactic features in this transfer learning setting.

### Transfer learning experiment

Table 2 presents the results of the TL study. For each fold of the cross-validation, we performed 10 experiments starting with different random weight initializations. Thus, each line of Table 2 is an average over 100 experiments. We observed that for the TreeLSTM model, additional source corpora consistently improved the performances. More interestingly, this phenomenon occurs even for corpora of distinct types of entities such as the combination of SNPPhenA and SemEval 2013 DDI and, to a lesser extent, with the corpus that is outside of the biomedical domain, reACE. To assess the statistical significance of the f-measure improvement obtained with the TL approach, we performed a Student’s t-test with a significance threshold *α* of 0.05. In every TL with SemEval 2013 DDI, the obtained *p*-values allow to reject the null hypothesis stating that there is no statistical difference between the two experiments: *p*-value(SNPPhena+SemEval)=4.5e-5; *p*-value(EU-ADR drug-disease+SemEval)=0.018; *p*-value(EU-ADR drug-target+-SemEval)=0.010; *p*-value(EU-ADR target-disease+-SemEval)=0.046. As a result, adding SemEval DDI as a source corpus improves performances over the baseline for all target corpora. We obtained higher *p*-values for the EU-ADR subcorpora, this could be explained by performance variability due to the small size of test samples associated with each class. We note that the pre-trained embeddings were obtained using biomedical sources. This may affect the TL performance with reACE that is not of the biomedical domain. Also, we did not observe any benefit of the TL strategy for the MCCNN model, which performances decrease slightly in comparison with the baseline experiments.

**Table 2:** Results of our TL strategy in terms of precision (P), recall (R) and f-measure (F)

Test Corpus	Model	Train corpus	P	R	F	*σ*_*F*_
SNPPhenA	TreeLSTM	SNPPhenA alone	58.9	73.8	65.5	4.1
		+ SemEval 2013 DDI	65.2	71.1	**68.0**	4.7
		+ ADE-EXT	62.8	72.1	67.2	3.4
		+ reACE	61.8	74.3	67.1	3.6
	MCCNN	SNPPhenA alone	55.1	75.0	**63.3**	4.8
		+ SemEval 2013 DDI	55.3	74.4	**63.3**	4.9
		+ ADE-EXT	56.1	73.2	63.2	4.8
		+ reACE	53.2	70.9	60.6	4.1
EU-ADR drug-disease	TreeLSTM	EU-ADR drug-disease alone	74.8	84.1	79.1	12.3
		+ SemEval 2013 DDI	74.8	90.6	82.0	13.1
		+ ADE-EXT	73.9	88.2	80.4	13.7
		+ reACE	74.3	91.1	79.3	14.3
	MCCNN	EU-ADR drug-disease alone	73.3	94.7	**80.2**	14.2
		+ SemEval 2013 DDI	72.6	87.9	76.6	14.3
		+ ADE-EXT	73.0	85.5	76.0	14.5
		+ reACE	74.1	91.5	79.2	13.8
EU-ADR drug-target	TreeLSTM	EU-ADR drug-target alone	72.4	90.6	80.2	10.9
		+ SemEval 2013 DDI	71.9	95.5	**82.5**	8.5
		+ ADE-EXT	70.2	96.7	80.9	9.2
		+ reACE	70.4	96.5	80.8	9.3
	MCCNN	EU-ADR drug-target alone	74.5	92.3	**81.0**	9.3
		+ SemEval 2013 DDI	74.9	88.8	80.0	10.6
		+ ADE-EXT	76.3	87.4	80.3	10.1
		+ reACE	73.4	92.1	80.5	7.8
EU-ADR target-disease	TreeLSTM	EU-ADR target-disease alone	77.0	89.7	82.7	6.4
		+ SemEval 2013 DDI	77.4	91.6	83.9	8.2
		+ ADE-EXT	77.7	89.5	83.3	6.9
		+ reACE	75.9	91.7	83.0	7.7
	MCCNN	EU-ADR target-disease alone	76.9	91.8	**82.6**	7.7
		+ SemEval 2013 DDI	77.6	90.6	82.5	7.1
		+ ADE-EXT	75.5	87.4	81.8	10.1
		+ reACE	77.1	91.2	82.0	6.8

*σ*_*F*_ is the standard deviation of the f-measure. The + in the column *Train corpus* indicates that we trained our model using the target corpus plus one additional source corpus. Bold numbers correspond to the best performing models

### Comparison with the state of the art

Table 3 presents a comparison of performances obtained with our approach *versus* two state-of-the-art systems applied to the RE tasks associated respectively with SNPPhenA [4] and EU-ADR [28]. Our results are obtained performing, for each fold, a unique experiment using an ensemble of the 5 best models for this fold, according to the experiments presented in Table 2. Ensembling is done by averaging the scores *s*(*r*_*s*_) of each individual model, following [15]. We reported the 10-fold average performance. Thus, each score in Table 3 is an average of 10 runs, one for each fold. Note that in the particular case of EU-ADR drug-disease, the ensembling does not lead to any improvement, which explains that performances reported are the same in Tables 2 and 3.. Both state-of-the-art systems use a combination of a shallow linguistic kernel with a kernel that exploits deep syntactic features. Our approach outperforms the performances reported for SNPPhenA and one EU-ADR subtasks and leads to similar performances for the two remaining EU-ADR subtasks.

**Table 3:** Performance comparison between the state of the art [4, 28] and this work in terms of precision (P), recall (R) and F-measure (F)

Test corpus	Work (train corpus)	P	R	F
SNPPhena	**[**4**]** (SNPPhenA)	56.6	59.8	58.2
	**This work** (SNPPhenA + SemEval 2013 DDI)	64.5	75.2	**69.4**
EU-ADR	**[**28**]** (EU-ADR drug-disease)	70.2	93.2	79.3
drug-disease	**This work** (EU-ADR drug-disease + SemEval 2013 DDI)	74.8	90.6	**82.0**
EU-ADR	**[**28**]** (EU-ADR drug-target)	74.2	97.4	**83.3**
drug-target	**This work** (EU-ADR drug-target + SemEval 2013 DDI)	73.5	95.6	83.1
EU-ADR	**[**28**]** (EU-ADR target-disease)	75.1	97.7	**84.6**
target-disease	**This work** (EU-ADR target-disease + SemEval 2013 DDI)	78.7	91.4	**84.6**

Results reported for this work are ensembles of the 5 best models obtained. Bold numbers correspond to the best performing models

### Analysis of the role of syntactic features in transfer learning

Empirical results suggest that the TreeLSTM model is more positively-influenced by syntactic similarity between source and target corpora than by domain closeness. Indeed, the TreeLSTM model explicitly includes the syntactic structure of the sentences in the network topology. Thus, a source corpus, such as reACE, that share neither entity nor vocabulary with the target corpus proved to be helpful. We propose in the following an analysis of the role of the syntactic features. We also provide real examples illustrating similarities between corpora and comment them.

**Fig. 4:**
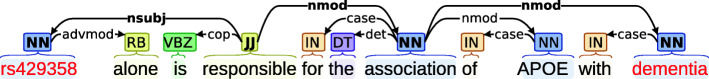
Dependency parse tree of a sentence from SNPPhena expressing a relation between the entities *rs429358* and *dementia*. The shortest dependency path between the two entities is shown in bold

**Syntactic features** We propose three comparisons based on patterns extracted from shortest paths between two entities in dependency graphs which link the two entities in relationship. Shortest path proved to be effective for RE [35,36]. From a shortest path (as between *rs429358* and *dementia* in Fig. 4), we extract 3 different patterns. The first one is made with the part-of-speech (POS) and dependency tags (DT): for example, in Fig. 4, "NN nsubj *JJ* nmod NN nmod NN"^2^. The second and the third patterns are built by keeping only either the POS or the DT. The patterns associated with our running example are then: "NN *JJ* NN NN" and "nsubj ** nmod nmod". For a given pattern, the *syntactic similarity* score is obtained using the following procedure: Given 2 corpora, (1) we first extract all the shortest path pattern that appear between two related entities. (2) For each corpus, we compute the pattern distribution (i.e., the list of patterns, along with their frequency) by normalizing over all the patterns in the corpus. (3) The score is then computed with the cosine similarity between the pattern distributions of two corpora. Table 4 shows the cosine similarity measures between target and source corpora for the three different pattern distributions. We observe that, for the two target corpora, the performance gain obtained with the TL strategy, using a given source corpus, can be related to the cosine similarity with this corpus: the higher cosine similarity leads to the best transfer TL.

**Table 4:** Cosine similarity score between target and source corpora for the three different pattern distributions

Target corpora	Source corpora		
	DDI	ADE	reACE
	POS + DT		
**SNPPhena**	0.53	0.22	0.13
**EU-ADR**	0.24	0.20	0.09
	POS only		
**SNPPhena**	0.80	0.70	0.35
**EU-ADR**	0.77	0.68	0.32
	DT only		
**SNPPhena**	0.53	0.23	0.14
**EU-ADR**	0.25	0.24	0.10

POS is part of speech pattern and DT is dependency type pattern

**Dictionary coverage** On the opposite, we observed that the efficiency of TL in our experiments can not be fully explained by the lexical similarity between source and target corpora. As shown in Table 5, the vocabulary overlap with the target corpora is almost equivalent whether we are considering DDI or ADE (53.4 *vs.* 51.2 and 58.9 *vs.* 60.5), whereas performances obtained with DDI were better than those obtained with ADE. Unsurprisingly, it is lower for reACE which is not a biomedical corpus.

**Table 5:** Dictionary coverage

	DDI	ADE	reACE
SNPPhenA	53.4	51.2	39.8
EU-ADR	58.9	60.5	38.3

Percentage of words from the target copora present in the source corpora

**Fig. 5:**
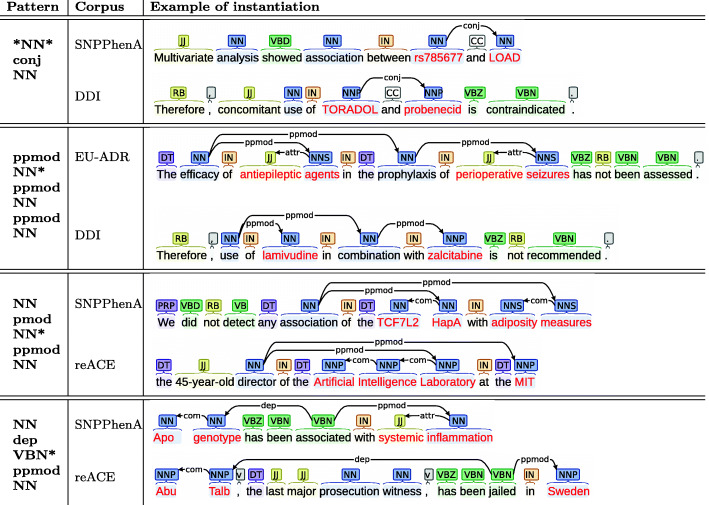
Examples of patterns and of their instantiation in corpora. Red words correspond to entities

**Lexical and semantic paradigms** We complete this analysis with few examples illustrating the lexical and semantic heterogeneity of sentences that may instantiate the same pattern. Figure 5 provides 4 patterns and their instantiations in source and target corpora. One can observe that sentences instantiating the same pattern seem to have no particular similarity when considering lexical and semantic paradigms. A similar heterogeneity is observed when considering the lowest common ancestor term (or the *head*) of the patterns. Table 6 lists the most frequent lowest common ancestor in each corpus. Again, we observe no direct link with learning improvement.

**Table 6:** Terms corresponding to the lowest common ancestor in the POS + DT patterns

SNPPhenA	EU-ADR	DDI	ADE	reACE
associated (25.2)	analyzed (5.8)	*entity* (17.8)	*entity* (30.1)	*entity* (60.6)
*entity* (12.2)	associated (4.3)	administered (4.1)	developed (11.1)	is (2.2)
genotyped (5.4)	*entity* (2.9)	increase (3.0)	associated (4.1)	was (1.9)
association (4.4)	is (2.9)	administration (2.7)	is (2.7)	said (1.4)
showed (3.8)	polymorphisms (2.4)	reported (2.6)	induced (2.3)	
observed (3.3)	over-represented (2.4)	interact (2.6)	case (1.6)	
genes (2.6)	showed (2.4)	reduce (2.5)	following (1.4)	

Their relative frequency in each corpus is provided in parenthesis. *Entity* means that the term is one of the two entities

## Discussion

This study empirically showed the impact of using syntax-aware models, in comparison with more classical convolutional models, for transfer learning. Since many high quality domain specific syntactic parsers are available (i.e. [37] for biomedical data or [38] for tweets), the proposed method can be used to improve performances for specific tasks for which few annotated resources are available.

The analysis using the proposed syntax-based metrics emphasizes the role of syntax in transfer learning using the TreeLSTM model. Several studies such as [39] and [40] have focused on selecting source data to improve transfer learning by preventing negative transfer. Future research should be done to leverage on the proposed metrics to guide the selection of additional training data. An exciting direction would be to explore this transfer strategy with Electronic Health Records of various origin.

## Conclusion

In this paper, we empirically showed that a TL strategy can benefit biomedical RE tasks when using the TreeLSTM model, whereas it is mainly harmful with a model that does not consider syntax. This is of great interest for specific domains, such those of biomedicine, for which few annotated resources are available. Our TL approach led *(i)* to better performances than the state of the art for two biomedical RE tasks: SNP-phenotype and drug-disease RE; and *(ii)* to state-of-the-art results for two others focusing on target-disease and target-drug relationships. Interestingly, we showed that even a general domain corpus (reACE) may carry useful information and lead to improved performances. We proposed an analysis with syntax-based metrics and examples to provide elements of interpretation of this behavior and emphasize the key role of syntax in TL for RE.

## Data Availability

All the corpus used in this study are publicly available and can be found at the following addresses: • SNPPhenA: https://figshare.com/s/b18f7ff4ed8812e265e8 • EU-ADR: https://biosemantics.org/index.php/resources/euadr-corpus • DDI: https://hulat.inf.uc3m.es/DrugDDI/DrugDDI.html • ADE: https://github.com/trunghlt/AdverseDrugReaction/tree/master/ADE-Corpus-V2 • reACE: https://catalog.ldc.upenn.edu/LDC2011T08

## Abbreviations

CNN: Convolutional Neural Network
DT: Dependency Tag
F: F-measure
LSTM: Long Short-Term Memory
MCCNN: MultiChannel Convolutional Neural Network
NLP: Natural Language Processing
NN: Neural Network
POS: Part of Speech
P: Precision
RE: Relation Extraction
R: Recall
RNN: Recursive Neural Network
SNP: Single Nucleotide Polymorphism
TL: Transfer Learning

## Footnotes

1 Macro f-measure gives the same importance to each class, while micro f-measure give the same importance to each sample. We used the macro f-measure because we don’t want to favor over-represented classes, but express the overall classification performances of our model across classes.

2 ^**2**^The stars mark the lowest common ancestor of the two entities in the dependency tree and are used to prevent similar pattern with different common ancestors to be considered the same. Note that the patterns are not directed, thus the two patterns *"NN nsubj *JJ* nmod NN nmod NN"* and *"NN nmod NN nmod *JJ* nsubj NN"* are equivalent.
